# Family and the field: Expectations of a field-based research career affect researcher family planning decisions

**DOI:** 10.1371/journal.pone.0203500

**Published:** 2018-09-07

**Authors:** Christopher D. Lynn, Michaela E. Howells, Max J. Stein

**Affiliations:** 1 Department of Anthropology, University of Alabama, Tuscaloosa, Alabama, United States of America; 2 Department of Anthropology, University of North Carolina Wilmington, Wilmington, North Carolina, United States of America; USC Keck School of Medicine, Institute for Global Health, UNITED STATES

## Abstract

Field-based data collection provides an extraordinary opportunity for comparative research. However, the demands of pursuing research away from home creates an expectation of unburdened individuals who have the temporal, financial, and social resources to conduct this work. Here we examine whether this myth of the socially unencumbered scholar contributes to the loss of professionals and trainees. To investigate this, we conducted an internet-based survey of professional and graduate student anthropologists (*n* = 1025) focused on the challenges and barriers associated with developing and maintaining a fieldwork-oriented career path and an active family life. This study sought to determine how (1) family socioeconomic status impacts becoming an anthropologist, (2) expectations of field-based research influence family planning, and (3) fieldwork experiences influence perceptions of family-career balance and stress. We found that most anthropologists and anthropology students come from educated households and that white men were significantly more likely to become tenured professionals than other demographic groups. The gender disparity is striking because a larger number of women are trained in anthropology and were more likely than men to report delaying parenthood to pursue their career. Furthermore, regardless of socioeconomic background, anthropologists reported significant lack of family-career balance and high stress associated with the profession. For professionals, lack of balance was most associated with gender, age, SES, tenure, and impacts of parenting on their career, while for students it was ethnicity, relative degree speed, graduate funding, employment status, total research conducted, career impact on family planning, and concern with tenure (*p* < .05). Anthropology bridges the sciences and humanities, making it the ideal discipline to initiate discussions on the embedded structural components of field-based careers generalizable across specialties.

## Introduction

*“Those same structures that have provided the resources for the academic disciplines to flourish have also restricted the means and content of knowledge production*.*”* Karri A. Holley (2013)

Despite calls for intersectionality in academia [1], structural challenges impede the success of women, those caring for dependents, and people of low socioeconomic status across disciplines at higher rates than other groups [2–6]. Intersectionality refers to the interconnected nature of social categorizations that apply to an individual or group, including race, class, gender, or status. However, these categorizations are also interrelated in systems of privilege and discrimination and challenge the very disciplines that study and seek to improve them. Among such challenges is family-career balance, which is a chronic concern in many professions and likely to affect those with least privilege [7,8]. For example, even in dual-parent working households, men spend more time at work than women and women more time on childcare and household chores [9–11]. This imbalance may contribute to the higher number of married women with children that are likely to leave their profession than men [12]. People from low-income families are also at a disadvantage in navigating academia as indicated by studies of first-generation college students, who are likely to come from such families. These students generally have less exposure to the options of graduate study, minimal awareness and knowledge of how university organizational structures work or fund students, and fewer people they see as potential academic mentors [13–15]. This lack of experience reinforces cycles of negative feedback in higher education [2,15–18]. Even among those for whom there are fewer structural impediments to success (i.e., white, cis-gendered men) [19], preparation for the demands of academic work-family balance are reportedly lacking [20]. These problems may be exasperated in anthropology, where a long history of field-based research expectations may produce additional barriers to recruiting a diverse workforce [21–24].

Advanced training in anthropology requires extensive research experiences that frequently include long-term immersive fieldwork in communities and organizations that necessitate extensive time away from home. In the 1920s, Malinowski’s “one man, one site, one year” framework for anthropological fieldwork transformed the discipline and modified expectations for trainees and professionals. Although this technique has significant benefits, it is an expensive (temporally and financially) and logistically complicated legitimizing process. Regardless, field-based research has become a paradigmatic rite of passage for challenging personal abilities [25,26].

For professionals, fieldwork provides space for ongoing and new research while playing a critical role in hiring, retention, promotion, and tenure. For instance, scholars with active field sites publish with greater frequency and receive more grants than those reliant on other strategies to conduct research [27]. The disciplinary importance of fieldwork relies on individuals who are socially unencumbered and financially solvent, either through their own means or external funding. However it systematically overlooks the significant social and financial responsibilities experienced by many professionals and trainees, including dependent family members (children, elderly parents, etc.), and household expenses (rent, car payments, student loan bills, tuition, credit card bills), and may act to systematically privilege those without these pressures. We explore whether this myth of the socially unencumbered scholar contributes to the loss of professionals and trainees in anthropology.

We test whether the expectation to conduct fieldwork in anthropology acts as a barrier that limits access by diverse socioeconomic, familial, and gendered backgrounds resulting in increased systematic homogeneity. Little attention has been paid to the pressures of family-career balance in anthropology with regard to fieldwork. To address how family-career balance and fieldwork affect scholars’ abilities to adhere to anthropology’s expectations, we investigated five intersecting questions related to the concept and practice of fieldwork as a discipline.

1. Is family socioeconomic status (SES) related to becoming an anthropologist?
2. Do expectations of anthropological fieldwork dissuade people from having children?
3. Do family responsibilities prevent those with or who would like to have children from entering the discipline?
4. Does having children impact individual ability to conduct fieldwork?
5. Does balancing family and anthropological careers influence perceived stress?

We pursued these questions through a survey of professional and graduate student anthropologists. These questions address the less obvious personal, social, and economic costs associated with fieldwork expectations of anthropology and examine the advantages and privileges that may indirectly support homogeneity in the profession.

## Methods

We used an online survey (Qualtrics, Provo, UT) with two waves of recruitment to examine the pressures of field-based research and perceptions of family-career balance in anthropology. The questionnaire included 73 items querying socio-demographic information, family planning, careers, children and fieldwork, and external family support in anthropology (S1 Appendix).

### Study recruitment

We administered the survey between April and November 2015 (*n* = 417) and between December 2015 and January 2017 (*n* = 722). We added the socioeconomic status (SES) questions in the second wave after preliminary analysis of survey qualitative responses suggested that anthropologists able to take children to the field came from higher socioeconomic backgrounds. We recruited English-speaking professional anthropologists and graduate students (both parents and non-parents) for participation using exponential non-discriminate snowball sampling through email, social media (Facebook, Twitter, Google+, Academia.edu), and flyers and presentations at professional meetings. We asked that these links to be shared by colleagues [28], while links to the survey were also provided on a department blog site and a post in an *Anthropology News* online column by the lead author [29,30]. The University of Alabama Institutional Review Board approved this protocol (#15-OR-134-R1).

### Assessing family support

To assess family influences and social support, we asked respondents if they were parents and, if so, what assistance/resources they have received around parenting. To determine SES, we used a modification of the MacArthur Scale of Subjective Social Status [31], which uses a pictorial ladder and asks respondents to indicate the rung that best represents their status with regard to education, income, and occupation. We modified the scaling from 10 rungs to 9 to group respondents in intervals of 3 as high (7–9), middle (4–6), and low status (1–3). We extracted a list of occupations from the Barratt Simplified Measure of Social Status [32] and also grouped those in association with high (7–9), middle (4–6), and low (1–3) economic status. We then created an SES variable for partners and parents by averaging education and occupation. To address structural impediments to raising a family while meeting the expectation of anthropological fieldwork, we asked respondents about support they received for parenting from partners, graduate advisers, colleagues, employers, and department chairs. We calculated overall support by summing these items. We measured family-career balance, impact of anthropology on family planning, and impact of family on anthropology careers using a series of items composed specifically for this study. Finally, we measured perceived stress using the 4-item Perceived Stress Scale [33].

### Statistical analysis

We downloaded both waves of survey data from Qualtrics and merged them in SPSS v.25 (IBM Corp., Armonk, NY). A total of 1135 respondents consented to participate and began the survey, while 4 dissented. We removed 110 incomplete responses for a final sample of 1025 participants. We generated descriptive statistics for all survey items and checked for errors and outlier values. In describing the data and testing hypotheses 1–4, we conducted bivariate analysis comparing professionals to students and women to men using *χ*^2^, Fisher’s exact, and independent samples *t* tests (there so were few non-binary gender respondents that we chose not include them in these comparisons). To test hypothesis 5, we conducted separate multiple linear regressions on perceived stress and family-career balance, retaining each as an independent variable in models of the other, using model variance to estimate the appropriate causal path. We chose other model variables based on bivariate correlations, theoretical considerations, and degrees of freedom. We standardized regression variables and tested for interaction effects among correlated variables of theoretical import (e.g., factors related to gender and minority disparities) but found none. Upon determining causal paths for professionals and students, we used AMOS V.25 (IBM Corp., Armonk, NY) to create visualizations of these models. We considered all statistics significant if *p* < .05.

## Results

### Respondent demographics

Table 1 outlines the demographics of the sample, the majority (80%) of whom identified as women. Most participants were living in (82%) and raised in (83%) North America and identified as white non-Hispanic (82%). For professional anthropologists, the mean age was 42.2 ± 9.54 (25–76) and for students 30.2 ± 6.62 (20–69). Most of the professionals (86.5%) and students (69%) were married or in committed relationships. Professionals were more likely to have at least one child (67%) compared to students (27%); and, among respondents with children, the mean ± SD was 1.3 ± .89 (1–5) for professionals and 1.6 ± 1.03 (1–8) for students.

**Table 1:** Demographics and comparison (χ^2^, Fisher’s exact) by career stage. Samples represented for each variable, and frequencies represent category, not full sample.

		Professionals		Students	
		*n*	%	*n*	%
Gender**	Women	366/488	75.0	421/497	87.7
	Men	121	24.8	72	14.5
	Genderqueer/liminal	1	0.2	4	0.8
Marital Status***	Married	354/488	72.5	186/497	37.4
	Committed relationship	68	13.9	158	31.8
	Single	40	8.2	137	27.6
	Separated, divorced, widowed	26	5.3	16	3.2
Children**	0	156/473	33.0	347/475	73.1
	1+	317	67.0	128	26.9
Ethnicity*	White	418/488	85.7	401/497	80.7
	Non-white	36	7.4	56	11.3
	No Response	34	7.0	40	8.0
**Region of residence**	North America (excluding Mexico)	385/476	80.9	406/492	82.5
	Outside North America	91	19.1	84	17.1
	Multiple countries	0	0	2	0.4
**Region of upbringing**	North America (excluding Mexico)	399/483	82.6	402/490	82.0
	Outside North America	78	16.1	76	15.3
	Multiple countries	6	1.2	12	2.4

*p < .05

**p < .01

***p < .001

Table 2 outlines demographic details specifically related to anthropology training. The largest single career demographic among respondents was the doctoral-level graduate student rank (34%), but professional academics (51%) and graduate students (49%) were evenly represented when collapsed into the two-group variable “career stage.” Nearly half (48%) were sociocultural anthropologists. As these ranks indicate, most respondents (49%) were employed full-time.

**Table 2:** Anthropological training, rank, and employment status and comparison (χ^2^, Fisher’s exact) by career stage.

		Professionals		Students	
		*n*	%	*n*	%
Highest Degree***	Doctorate	462/487	94.9	10/494	2.0
	Master’s	23	4.7	399	68.6
	Bachelor’s	2	0.4	145	29.4
Training**	Sociocultural	255/475	53.7	207/477	43.4
	Biological	88	18.5	105	22.0
	Archaeology	68	14.3	67	14.0
	Applied	43	9.1	83	17.4
	Linguistics	14	2.9	11	2.3
	Other	7	1.5	4	0.8
Employment status***	Full-time	397/486	81.7	78/496	15.7
	Underemployed	69	14.2	94	19.0
	Unemployed	5	1	22	4.4
	Full-time, funded student	7	1.4	301	60.7
	Retired	8	1.6	1	0.2
**Career rank**	Professors emeriti	8/488	1.6	-	-
	Professor	59	12.1	-	-
	Associate professor	113	23.2	-	-
	Assistant professor	125	25.6	-	-
	Lecturer	50	10.2	-	-
	Adjunct	55	11.3	-	-
	Postdoc	78	16.0	-	-
	Doctoral student	-	-	339/497	68.2
	Master’s student	-	-	158	31.8

*p < .05

**p < .01

***p < .001

**Fig 1:**
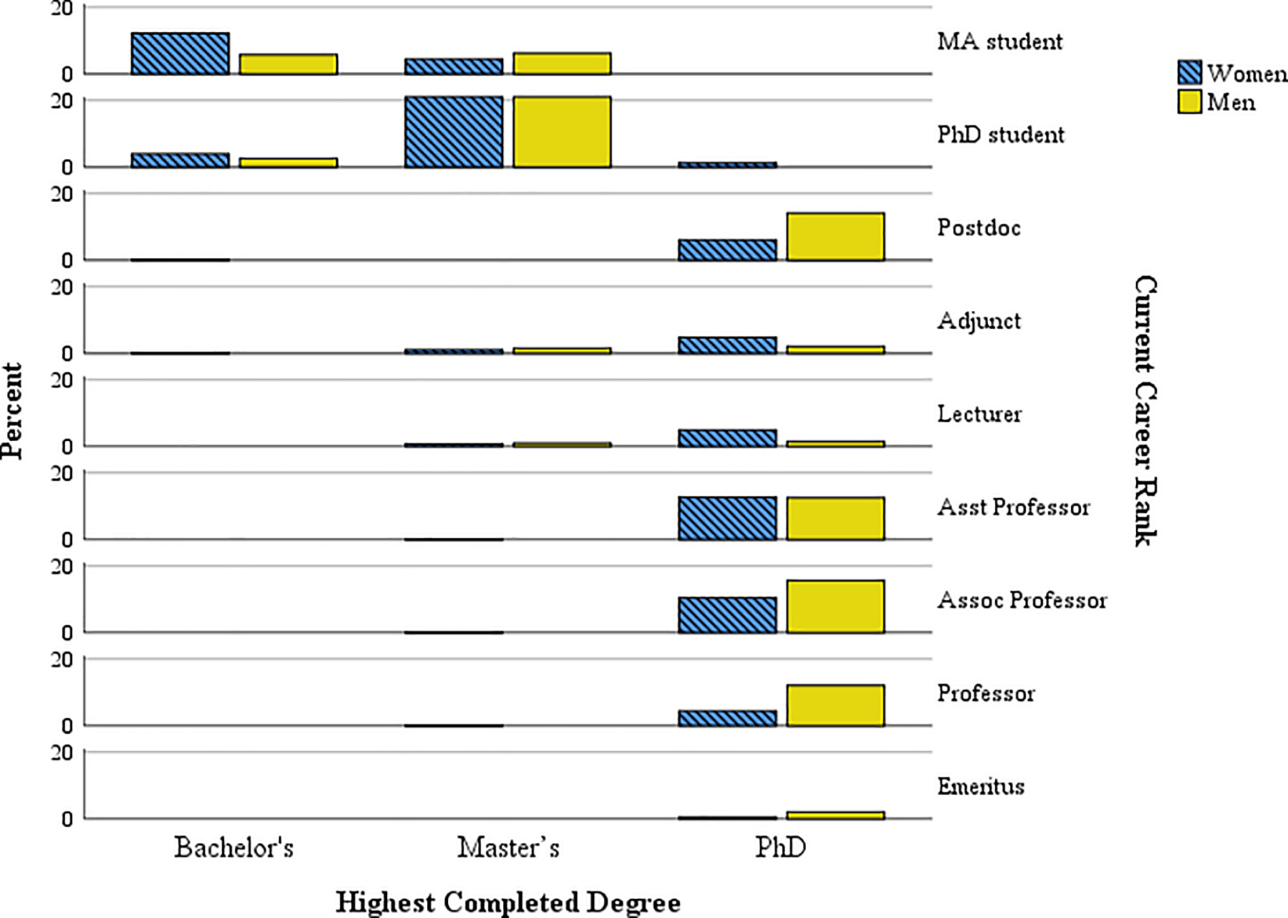
Percentage of women and men by highest degree completed.

We compared degree rates and employment of women and men to assess whether there were disparities consistent with the professional attrition of women in other studies [3–6,23,24]. Fig 1 illustrates this comparison, indicating significantly (*p* < .01) higher percentages of women among MA and PhD students and PhD-holding adjuncts and lecturers but higher percentages of men among PhD-holding tenure-track and tenured professors (42%). There were also more men (*p* < .01) among those with full-time employment (59%). Since this is a convenience sample, it is unknown if these rates are representative of the discipline of anthropology or if there was differential dropout based on differing interests in aspects of the study.

### Family socioeconomic background

We predicted that people from an educationally privileged background would be more able to pursue an anthropological career. We tested this by eliciting information about spousal and family education and, in the second survey wave (*n* = 573), personal (1 = lowest, 9 = highest), spousal, and family socioeconomic self-ratings (low/middle/high). As outlined in Table 3, the majority of our study participants came from highly educated families, wherein at least one parental figure had a bachelor’s degree or higher (72%), and about half had at least one parent who completed a graduate degree. There were no differences between women and men or professionals and students in family educational background. Mean ± SD (min-max) SES for professional respondents was 6.62 ± 1.35 (1–9) and for students was 5.87 ± 1.51 (2–9). The majority of respondents’ partners (76%) and parents were high status (61% for mothers and 70% for fathers).

**Table 3:** Socioeconomic status of respondents, partners, and parental figures and comparison (χ^2^, Fisher’s exact) by career stage.

		Professionals		Students	
		*n*	%	*N*	%
**Family Education**	Doctorate	94/488	19.9	78/496	15.7
	Master’s	156	32	142	28.6
	Bachelor’s	105	21.5	126	25.4
	Some college	46	9.4	54	10.9
	First generation	84	17.2	96	19.4
Perceived SES**	High	190/300	63.3	108/262	41.2
	Middle	100	33.3	132	50.4
	Low	10	3.3	22	8.4
**Partner SES**	High	234/301	77.7	106/136	77.9
	Middle	59	19.6	29	21.3
	Low	8	2.7	1	0.7
**Parents’ SES**	High	216/362	59.7	87/149	58.4
	Middle	130	35.9	55	36.9
	Low	16	4.4	7	4.7

*p < .01

**p < .001

### Fieldwork and family planning

To assess how an anthropology career may impact family planning, we asked respondents with children about family structure, planning of children, career stage when children were born, and the aspects of anthropology that most influenced family planning. Most were part of two-parent nuclear family units (including stepparents) (85%) (Table 4), and the majority had their first child during graduate school (38%). Eighteen percent had a first child before graduate school and another 18% had a first child after graduate school but before obtaining a full-time permanent position. Fifteen percent of respondents had a first child during the pre-tenure period of academic employment, while 5% had a first child post-tenure. A second child was also most likely to be born during graduate school (17%), with 13% born during the pre-tenure period of tenure-track employment, 10% born between graduate school and full-time employment, 6% born before graduate school, and 5% born after obtaining tenure.

**Table 4:** Family planning and impact of anthropology career and comparison (χ^2^, Fisher’s exact) by career stage.

		Professionals		Students	
		*n*	%	*N*	%
**Career impact on family planning**	High	11/148	7.4	39/328	11.9
	Moderate	43	29.1	95	29.0
	Minimal	59	39.9	120	36.6
	None	35	23.6	74	22.6
Future plans to become parent#**	Likely	54/152	35.5	176/338	52.1
	Unsure	24	15.8	93	27.5
	Unlikely	33	21.7	33	9.8
	Will not	41	27.0	36	10.7

#Future plans to become parent was only queried among respondents with no children.

*p < .01

**p < .001

**Fig 2:**
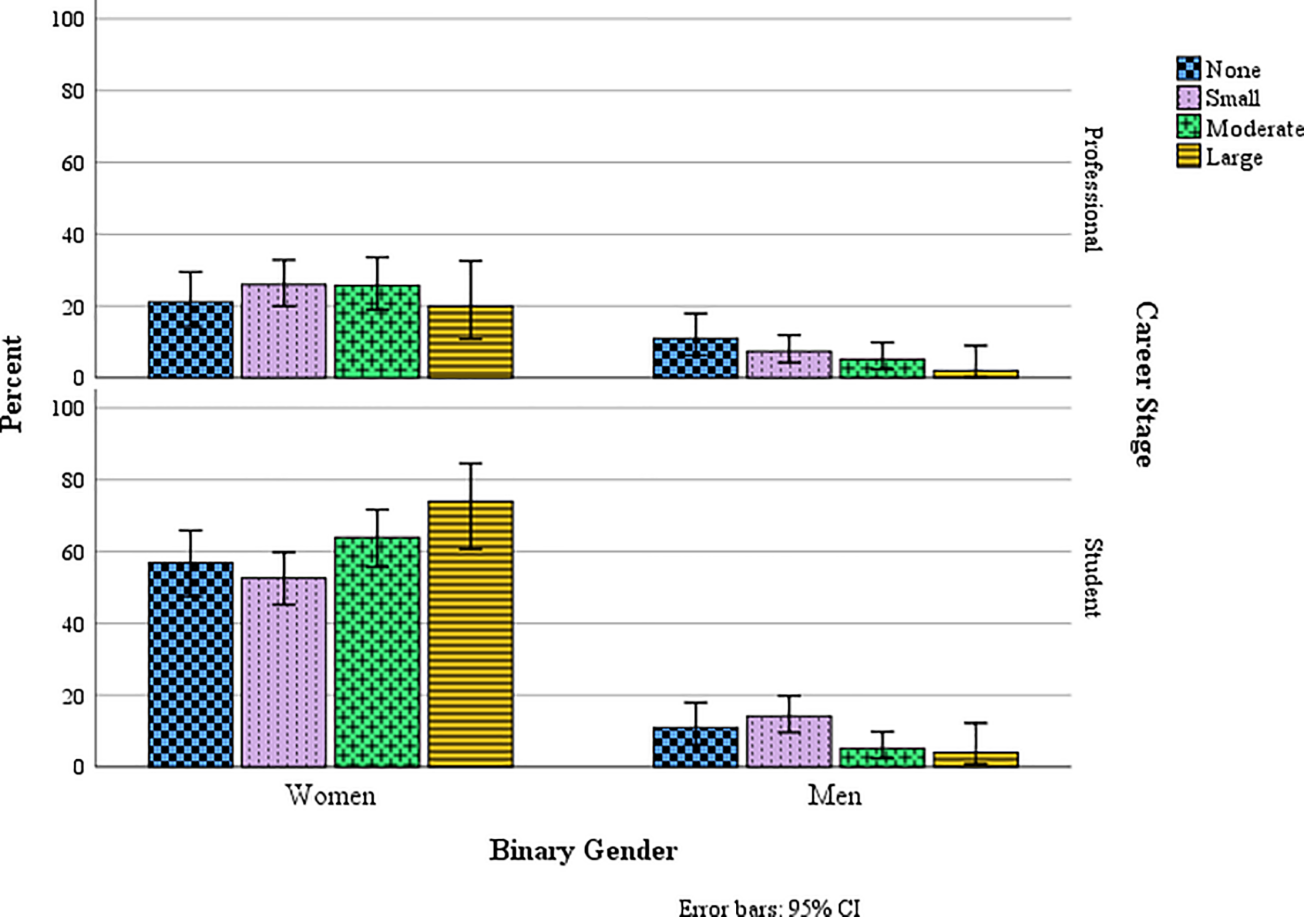
Impact of anthropology career on family planning for non-parents by career stage and binary gender (relative %).

**Fig 3:**
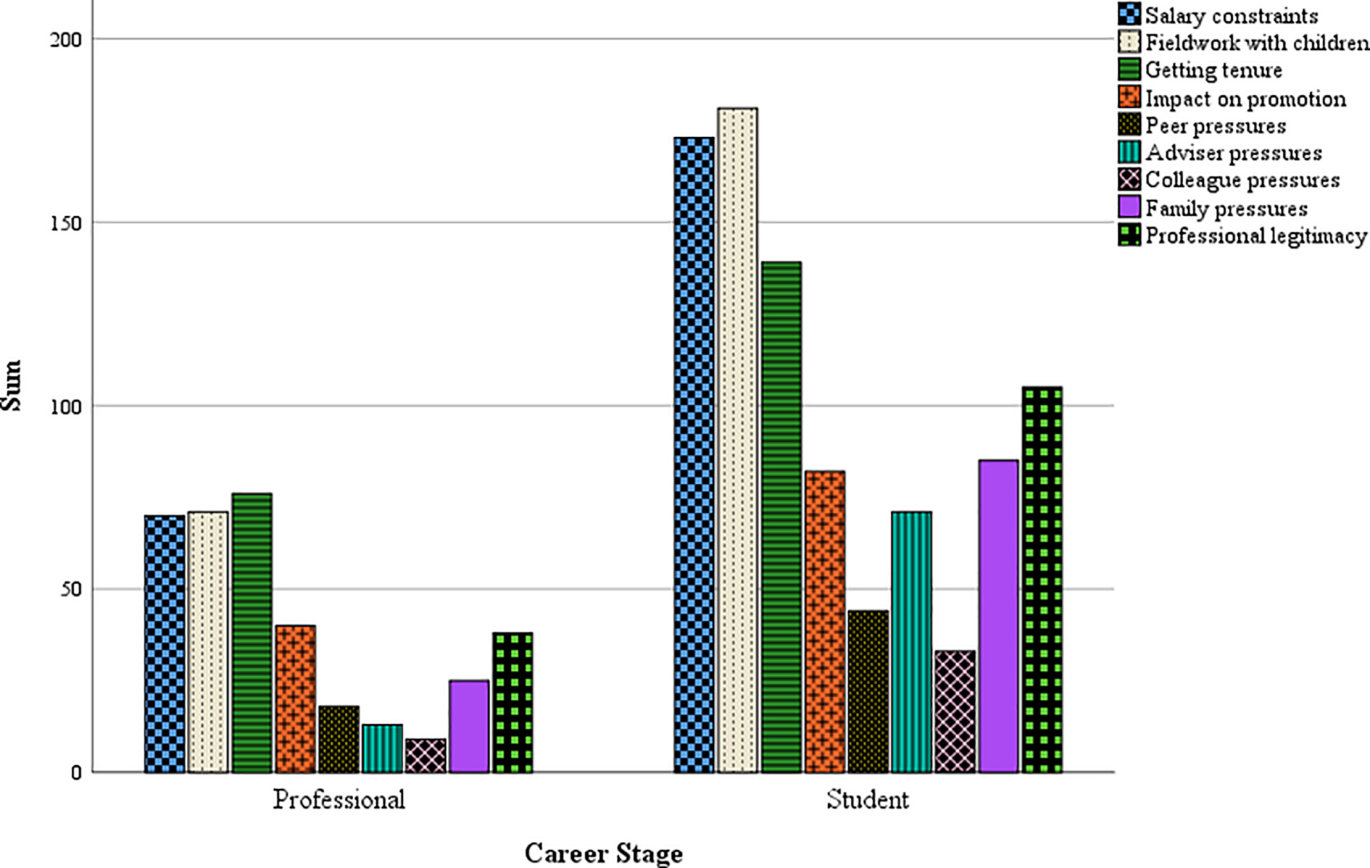
Factors influencing expectations of career in anthropology by career stage.

More than half of respondents without children (60%) indicated that their career in anthropology had impacted their decision to not have children. Family planning decisions had greater impacts on women than men for parents and non-parents in our sample (Fig 2), a difference that was significant among students (*p* = .003) but not professionals (*p* = .057). Family planning decisions of women were significantly more likely to be affected by concerns with conducting fieldwork, getting tenure, impacts on promotion, preconceived notions of peers, and disappointing their advisors than in men (*p* < .05). Students were significantly more concerned than professionals with all aspects of anthropological careers that could affect family planning (*p* < .01). Among the concerns, professionals and students alike were most concerned with salary constraints, conducting fieldwork with children, and getting tenure (Fig 3).

### Children and fieldwork

We predicted that anthropologists without family resources to care for children during fieldwork would reduce or cease fieldwork upon becoming parents. Regardless of gender or career stage, the majority of those with children (56%) indicated that parenthood did not impact their decision to pursue a career in anthropology. Women and men tended to go to distant sites (defined as sites that required travel away from home for multiple overnight stays) to conduct fieldwork at least every few years (64%). The mean number of months parents spent in the field conducting research per field season was .76 ± 1.578 (0–15.6) or approximately 3 weeks.

To address whether having children impacted anthropological fieldwork, we queried patterns of fieldwork after having children, how children were cared for, and the quality of experiences, as outlined in Table 5. The majority of respondents with children (80% professionals, 71% students) had been to the field to conduct research since becoming parents. This postpartum fieldwork was significantly more likely among men (86%) than women (74%, *p* = .02). Most respondents with children (56%) had never taken a child to the field, and there were no gender differences among those who had done so.

**Table 5:** Field experiences with children and comparison (χ^2^, Fisher’s exact) by career stage.

		Professionals		Students	
		*n*	%	*n*	%
Travel to distant field site**	Multiple times per year	56/233	24.0	13/84	15.5
	Annually	53	22.7	13	15.5
	Every few years	61	26.2	6	7.1
	Few times in career	38	16.3	23	27.4
	Once ever	10	4.3	7	8.3
	Never	15	6.4	22	26.2
Where children stay during remote fieldwork*	Come to field site	37/254	14.6	18/98	18.4
	With co-parent	128	50.4	23	28.6
	With grandparent	3	1.2	5	5.1
	With another relative	0	0	2	2.0
	With a non-relative	5	2.0	3	3.1
	Combination	81	31.9	42	42.9
**Quality of experience when taking child(ren) to field**	Good for child(ren) and productive for research	72/162	44.4	17/48	35.4
	Good for child(ren) but difficult financially/productively	72	44.4	23	47.9
	Difficult for child(ren) but productive for research	7	4.3	5	10.4
	Unproductive for child(ren) and research	11	6.8	3	6.3

*p < .01

**p < .001

Women and men used a variety of resources for childcare while in the field, though men tended to rely exclusively on a co-parent or combination of childcare options, whereas women more often utilized grandparents and non-relatives (*p* = .01). The majority of those who had taken their kids to the field reported it as a good experience for the children (87%), though half (51%) also reported that it made fieldwork more difficult.

### Balancing family and an anthropological career

**Fig 4:**
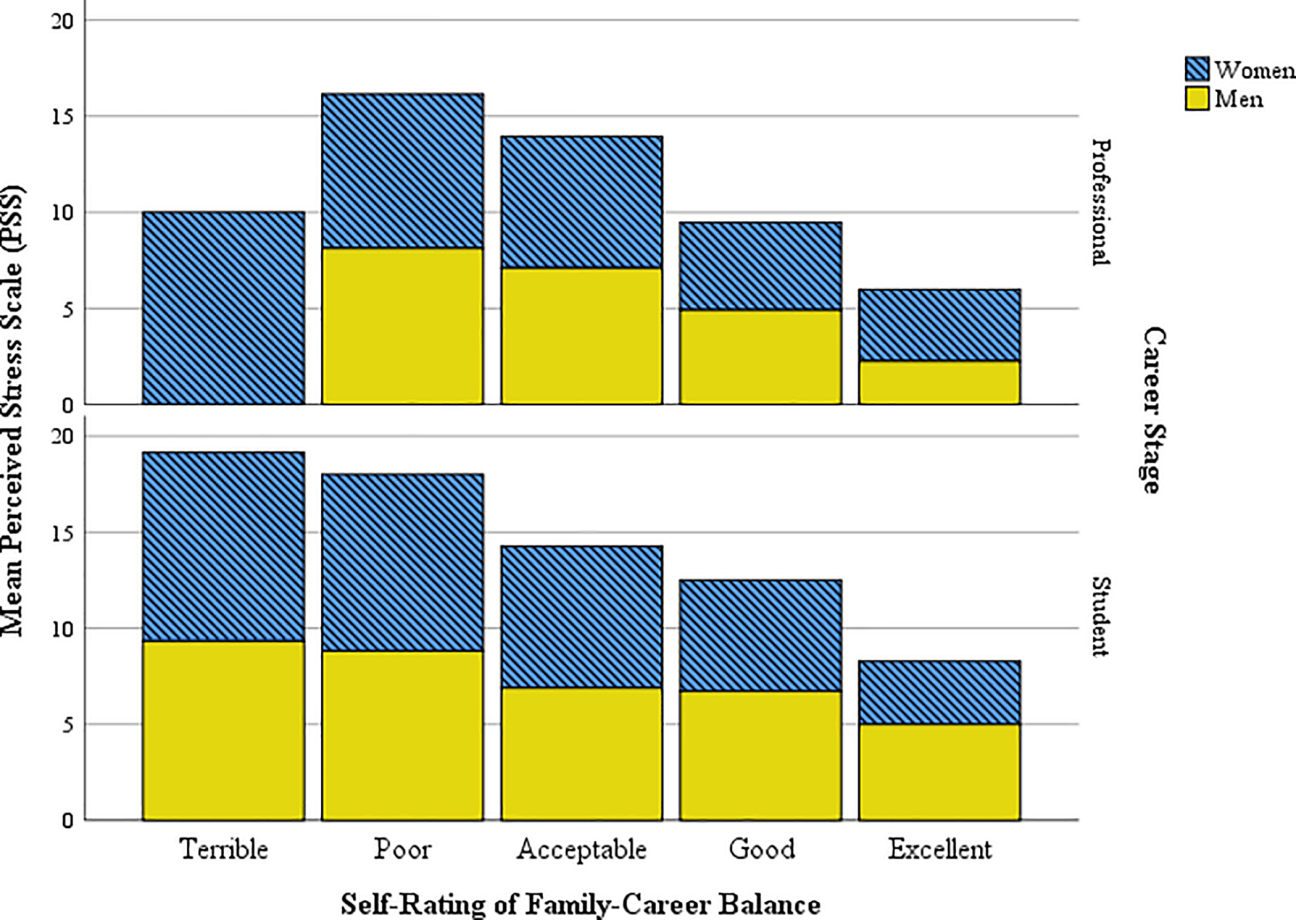
Self-rating of family-career balance against perceived stress by binary gender and career stage.

To determine how anthropologists handle the pressure of work and family, we asked participants about general perceived stress and self-rating of family-career balance. On a 5-point scale (1 = terrible, 5 = excellent), the rating valence for professionals (3.1 ± .04) was significantly more positive than students (2.8 ± .04, *p* < .001). Among professionals, men (3.3 ± .91) were significantly more positive than women (2.9 ± .03, *p* = .001), whereas there was no difference by gender among students (Fig 4). Similarly, perceived stress was significantly higher among students (7.7 ± 3.0) than professionals (6.5 ± 3.0, *p* < .001), but there were no gender differences in perceived stress.

To reflect the differing pressures experienced by professionals and students, we conducted separate bivariate correlations to select variables for regressions on perceived stress and family-career balance. We considered findings to preceding hypotheses and, for professionals, chose variables that were significantly correlated with perceived stress, family-career balance, mean time in the field, or SES. To ensure sufficient degrees of freedom, we included only variables with *n* ≥ 250 (Table 6).

**Table 6:** Bivariate correlations for professionals of variables *n* ≥ 250.

	Perceived stress	Family-career balance	Mean time in field	SES
Gender	-0.056	.153**	0.062	-0.030
Age	-.231**	.215**	.467**	.174**
Marital status	-0.064	.128**	.193**	0.088
Partner in academia	-0.050	0.012	0.046	0.086
Have children	-.121**	.118*	.484**	.139*
Ethnicity	-0.051	0.050	0.087	0.051
Country raised in	0.009	0.034	0.015	0.056
Spouse SES	-0.010	0.045	0.009	0.108
Parents SES	-0.016	0.057	0.057	-0.019
Family education background	-0.015	0.074	0.089	.223**
Time to complete highest degree	0.085	-0.016	0.058	-0.010
Anthropological training	0.019	0.001	-.129**	-0.067
Relative graduate funding	-.125**	0.080	0.036	0.095
Current employment status	-.209**	.132**	0.081	0.110
Total research conducted	-0.070	0.063	.276**	0.018
Financial obligations to dependents	0.071	-0.049	-0.066	-0.080
Institutional support for family	-0.085	0.062	-0.048	0.085
Parenting impact on career	.146*	-.196**	-.142*	-.166*
Supervisor gender	-0.046	0.065	-0.017	0.088

* *p* < .05

**p < .01 (2-tailed).

We constructed separate linear regression models for perceived stress and family-career balance, including each as independent variables for the other to determine causality. For professionals, the regression on perceived stress including family-career balance as independent variable explained more variance (*F*_12,169_ = 6.880, *p* < .001, *r*^2^ = .328) than did regression on family-career balance with perceived stress as independent variable (*F*_12,169_ = 6.404, *p* < .001, *r*^2^ = .313) (Table 7).

**Table 7:** Multiple regressions on perceived stress and family-career balance for professionals.

	Perceived stress		Family-career balance	
	*ß*	*p*	*ß*	*P*
Gender	.034	.602	.109	.097
Age	-.084	.278	.065	.407
Marital status	.036	.585	.063	.344
SES	-.137	.044	.012	.858
Family education background	.056	.393	.046	.487
Anthropological training	.007	.911	.006	.932
Relative graduate funding	-.068	.290	.011	.861
Current employment status	-.114	.086	-.012	.864
Total research conducted	.009	.896	-.007	.925
Parenting impact on career	.008	.904	-.110	.100
Mean time in field	-.031	.673	-.018	.810
Family-career balance	-.469	< .001		
Perceived stress			-.480	< .001

Because of the significant effect of family-career balance on perceived stress, we regressed several dummy variables related to things that lead to delaying or avoiding parenthood among non-parent respondents (Table 8), including concerns about salary, balancing children and fieldwork, tenure, promotion, peer pressures, advisor pressures, colleague pressures, and family pressures. We retained gender, age, marital status, and parent status as controls. We found that gender, SES, having children, and concerns with tenure were significant influences on family-career balance.

**Table 8:** Multiple regression on family-career balance among professionals.

	*ß*	*p*
(Constant)		< .001
Gender	127	.025
Age	132	.030
Marital status	.094	.128
SES	.145	.013
Have children	-.182	.027
Salary	-.002	.978
Fieldwork challenges	.029	.728
Tenure	-.310	.001
Promotion	-.057	.459
Peer pressures	-.018	.794
Adviser pressures	-.004	.956
Colleague pressures	.033	.628
Family pressures	.002	.970

**Fig 5:**
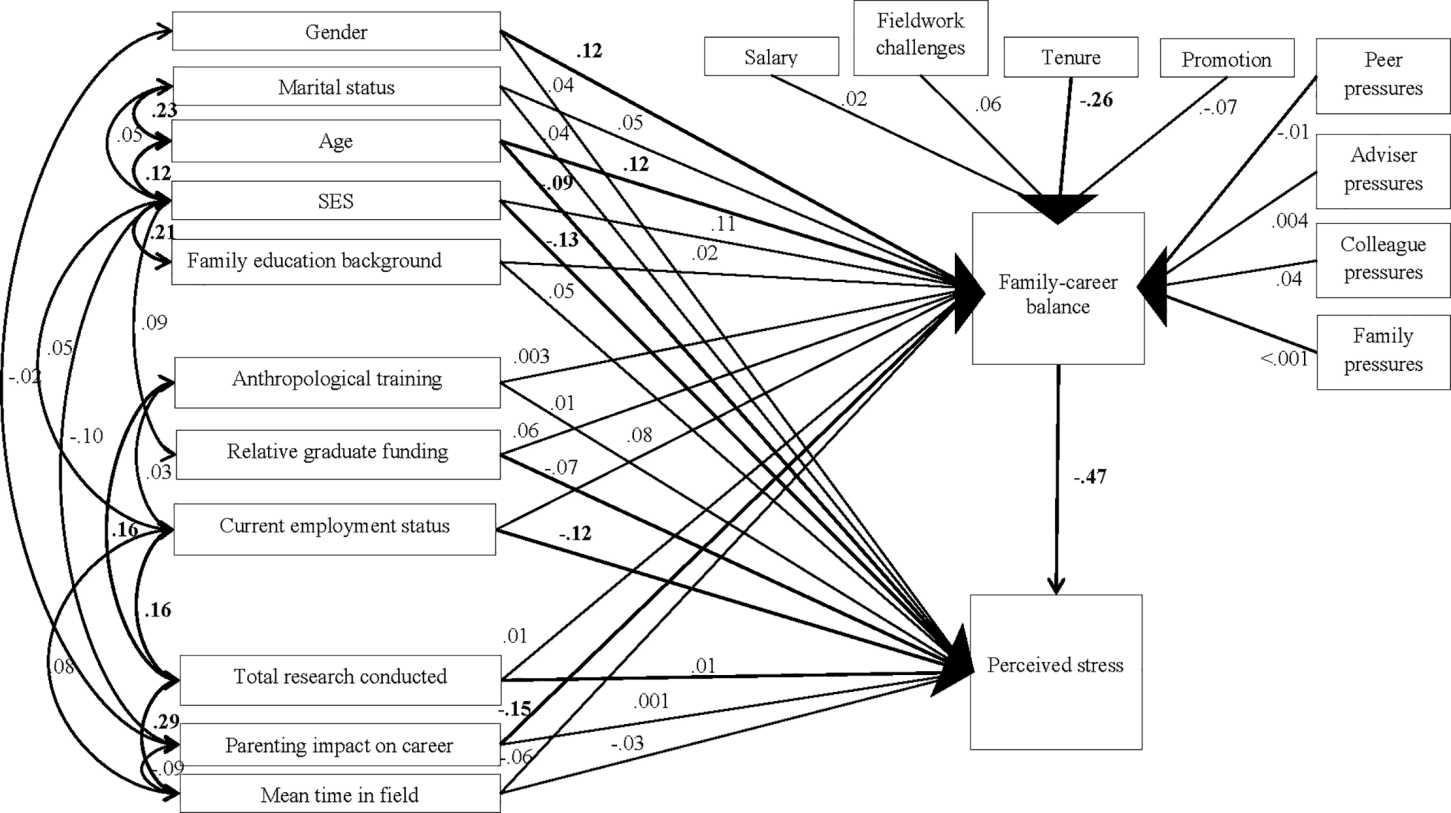
Path analysis of family-career balance and perceived stress among professionals.

To put these models in context, we constructed a path model as illustrated in Fig 5. Of note in this model are the significant influences of parenting on career and concerns around tenure that caused people to forgo or delay having children.

Most students are not expected to have completed fieldwork, so we examined bivariate correlations with respect to perceived stress, family-career balance, and SES only and included other variables relevant to students (Table 9).

**Table 9:** Bivariate correlations for students of variables *n* ≥ 250.

	Perceived stress	Family-career balance	SES
Gender	.017	-.063	-.101
Age	-.073	.033	.097
Marital status	-.043	.163**	.018
Partner in academia	.117*	-.001	.024
Country resides in	-.049	.044	.092
Family education background	-.027	-.004	.319**
Highest degree completed	.033	-.034	.132*
Time to complete highest degree	-.029	-.018	.027
Relative speed of highest degree	-.002	.094*	-.052
Anthropological training	.012	-.031	-.061
Graduate funding	-.014	-.093*	.055
Graduate stipend	-.072	-.024	.084
Relative graduate funding	-.059	.028	.035
Currently employment status	-.107*	.056	-.111
Total research conducted	.033	-.114*	-.060
Plans for future children	-.161**	.100	.187**
Plans regarding children or career	-.029	.018	-.063
Impact of career on family planning	.125*	-.275**	-.047

*p < .05

**p < .01 (2-tailed).

As with professionals, we conducted separate regressions among student respondents on perceived stress and family-career balance, including each as independent variables for the other (Table 10). Unlike professionals, we found that the model predicting student family-career balance (*F*_12,173_ = 6.421, *p* < .001, *r*^2^ = .308) explained more variance than that predicting perceived stress (*F*_12,173_ = 5.739, *p* < .001, *r*^2^ = .285).

**Table 10:** Multiple regressions on perceived stress and family-career balance among students.

	Perceived stress		Family-career balance	
	*ß*	*P*	*ß*	*p*
(Constant)		< .001		< .001
SES	-.214	.004	.078	.285
Partner in academia	.119	.071	.015	.813
Ethnicity	-.051	.444	-.117	.070
Country raised in	.028	.673	-.027	.681
Family education background	.039	.573	-.048	.482
Relative degree speed	.001	.986	.102	.120
Graduate funding	-.036	.582	-.104	.109
Current employment status	-.115	.090	.015	.822
Total research conducted	-.016	.807	-.082	.207
Plans for future children	-.103	.134	.055	.416
Career impact on family planning	.013	.847	-.227	.001
Family-career balance	-.405	< .001		
Perceived stress			-.392	< .001

We tested dummy variables related to delaying or refraining from having children against perceived stress among students, controlling for SES and having a partner in academia, but only SES was a significant predictor (Table 11).

**Table 11:** Multiple regression on perceived stress among students.

	*ß*	*p*
(Constant)		< .001
SES	-.271	< .001
Partner in academia	.130	.069
Salary	.031	.741
Fieldwork challenges	-.041	.667
Tenure	.174	.088
Promotion	-.040	.688
Peer pressures	-.053	.519
Adviser pressures	-.069	.447
Colleague pressures	.086	.334
Family pressures	.044	.598

**Fig 6:**
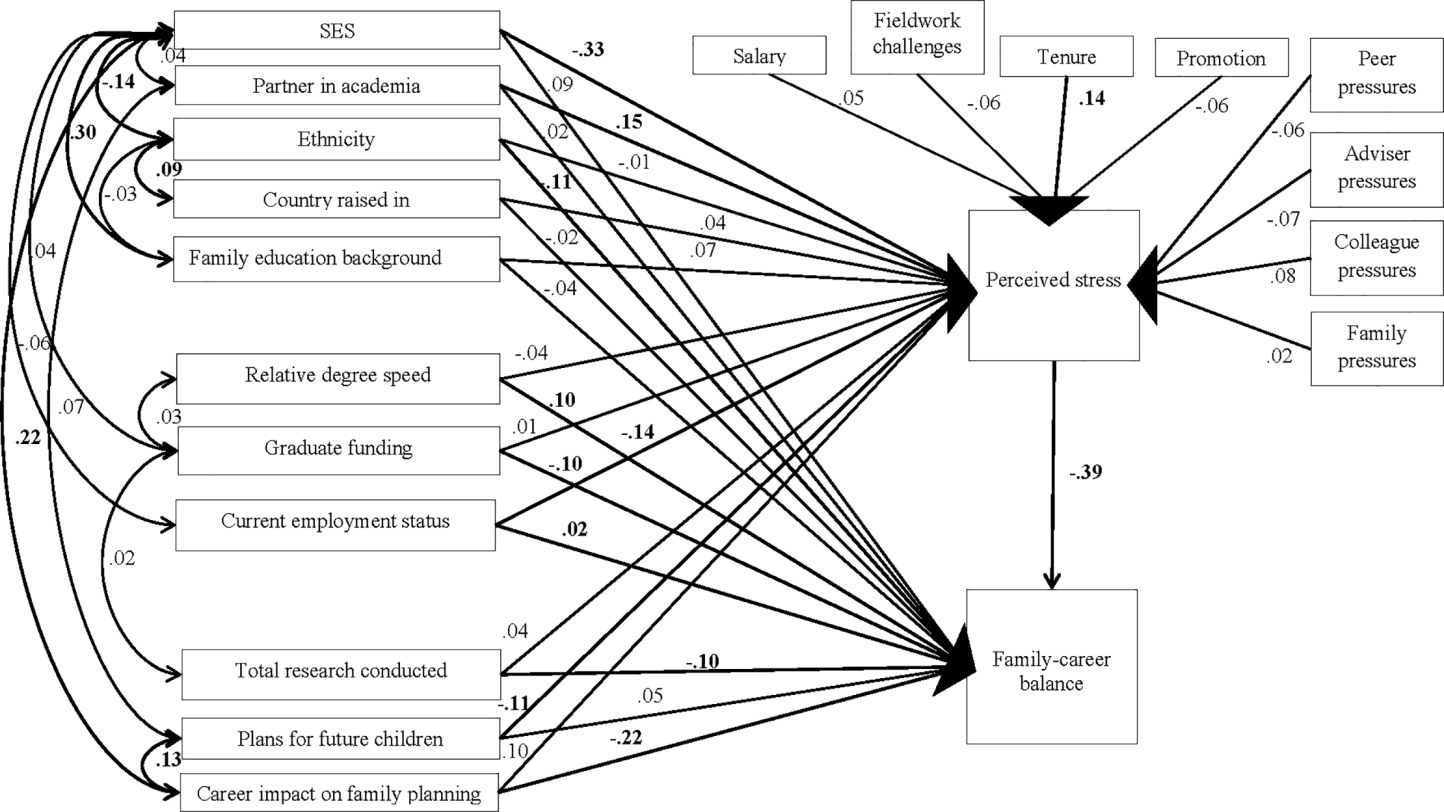
Path analysis of perceived stress and family-career balance among students.

Finally, we created a visualization of the path model for students (Fig 6). Where separate regressions indicated on an influence of SES and family-career balance on perceived stress and career impact on family planning on perceived stress and the sense of family-career balance, the path model indicates several other salient influences. Having a partner who is also in academia significantly increases stress, as do negative employment status and, curiously, planning not or being unsure about future children. Among students, being white was significantly associated with a positive sense of family-career balance, as was positive employment status. There was a significant relationship between a low career impact on family planning and a positive sense of family-career balance. In theory, students have yet to conduct much research, so it may be consistent that there was a significant negative relationship between total research conducted and family-career balance, though the significant negative relationship between funding and family-career balance is unclear. Despite being students, delaying or refraining from parenthood because of concerns with tenure is as great a concern as among professionals.

## Discussion

Anthropology is a field-based discipline that utilizes a comparative approach to understand humanity. However, social and financial barriers may undermine intersectionality in the discipline and prevent some individuals from pursuing an anthropological career. We examined perceived stress and family-career balance among anthropologists and those training to become anthropologists with regard to SES, gender, and family planning decisions. To accomplish this, we used a convenience survey.

### Women in anthropology

In anthropology, women are more likely to enroll in undergraduate and graduate courses and are represented in higher numbers among young professionals [23]. This is consistent with other social science fields, but in opposition to fields considered more “mathematically intensive” [34]. Our results indicate that representation at earlier stages doesn’t necessarily mean equality in later career stages. Women in our study were less likely to hold PhDs, professional rank, or full-time employment. Our study also indicated that a career in anthropology had a more negative impact on women’s family planning than on men’s across an array of domains, including childcare, academic advisor support, tenure and promotion, equity in salary, and attitudes of peers. Women in this study were more likely to perceive themselves as disadvantaged concerning structural (salary, fieldwork plans, career plans, social pressure) and social constraints (opinions of family, colleagues, or superiors). They also ranked their family-career balance more negatively than men. These findings reflect other studies of family-career balance in academia, which find that women, particularly those who are pre-tenure, report more difficulty juggling parental and work responsibilities than men [35]. For instance, women scientists report sacrificing discretionary or leisure time and flexibility more than men [35–37]. Loss of leisure time is associated with increased stress, while loss of flexibility can be directly related to reducing abilities to travel for work. These reductions negatively influence opportunities for collaboration and recognition for research [35,38]. As others [12,23] indicate, these factors are directly responsible for women leaving academia at a substantially higher rate than white men. A 35-year-old female PhD student respondent to our study confirmed this, indicating,

academia showed me the people I once respected as leading researchers had no work life balance and had sacrificed the other elements of life, like family, to work 100-hour weeks to excel in [the] field [of anthropology]. Working hard is one thing but that attitude of being the ONLY way to be a quality scientist turned me off the profession entirely.

For those pursuing a graduate degree or tenure, the demands of children can slow their progress and thus represent a significant trade-off in family-career balance [39]. This is a particular challenge to women, whose children are physically dependent on them during pregnancy, birth, and breastfeeding and who may suffer punitive policies that limit postpartum rehabilitation. A white 49-year-old tenured female professor stated,

I had to fight, hard, to get any maternity leave. I was fighting so much that my senior colleagues (all male except for one) turned me down for early tenure. I ended up taking an extra year on my clock to let the whole mess blow over. I came up for regular tenure late, got it with a unanimous vote. Guess my colleagues wanted to say fuck you without getting sued. It worked!!

Several women addressed this challenge when it came time for fieldwork by taking their children with them because they simply had no other choice. As a 31-year-old female student indicated she took her child “because he was a baby and breastfeeding for the first time. Because I want to be with him, when he grew up.” Similarly, another female student (age 35) stated,

both my bio kids nursed well into toddlerhood. They needed me to sleep and pumping for any long amount of time just wasn’t working for our family. Also, my husband’s schedule isn’t very flexible so childcare was easier to arrange if the kids were with me. Also, fuck that broken family shit. I just wanted my babies with me. I like ‘em.

For many, the response of colleagues, academic advisors, and supervisors was crucial to their perceived balance. However, this support ranged from very positive (e.g., advisors who hosted baby showers) to very negative (“My dissertation advisor was nearly emotionally abusive when I became pregnant while writing my dissertation”). One woman who had achieved tenure shared, “I keep my kids a total secret in academia”.

In addition, women were less likely to have conducted field-based research since having a child. When they did, women were dependent on support from their parents more than their male peers were (“I have taken them when I have had a grandparent with me who can take off work. Otherwise, I have not taken them with me.”), who were more dependent on spousal support (“Child was very young and fieldwork was several months long, so wife and daughter came along.”). Support from family and academic peers has a significant impact on individual abilities to conduct extended stretches of fieldwork, the places where fieldwork can be conducted (safety, distance, etc.), and possibly the quality of the work that can be conducted, which echoes findings on family-career balance in academia in general [35]. A 28-year-old white female student said, “my step-daughter and partner are Tanzanian, which is where I conduct my fieldwork. Therefore, they are in the same country but don’t go with me to my particular data collection sites.” By contrast, another student (36-year-old, white female) opted to do research in the US, stating, “I chose to set up a domestic project so that I could have children. The site is two hours from my home. I frequently brought my infant son with me; it was very difficult to bring him or leave him.” Finally, few grant agencies permit grantees to use funding to support childcare or travel for family members, which creates additional financial restrictions on fieldwork plans. Taken together, this can have cumulative, long-term effects on women’s careers and act as a barrier to promotion [12].

Gender based barriers have been identified in other field-based disciplines, including science, technology, engineering, and mathematics (STEM) fields. Recent studies find that masculine culture is overvalued in field settings when women are excluded [40,41]. In general, women and minorities in STEM are severely underrepresented, have lower rates of retention, and are most likely to switch their majors to non-STEM fields [42]. Since Marie Curie won a Nobel Prize in 1903, only 17 other women have received the award in the areas of physics, chemistry, or medicine compared to 572 men, and only 28% of the world’s researchers are women [43]. Analysis of adolescent achievement in STEM careers internationally indicates higher performance among girls than boys in two-thirds of sampled countries but an inverse relationship between “national gender equality” and pursuit of STEM degrees by women [44]. Our research supports that this difference may be driven in part by hidden barriers associated with a mismatch between family and career expectations that may result in the reduction of qualified individuals from research fields.

### Family and the field

Many participants in our study indicated that they delayed having children or avoided taking them to the field because of fears related to professional credibility or disapproval of an adviser or supervisor. However, we found no gender differences in opinion on whether taking children to a field site is a positive or negative experience. Some people found that taking their children to the field was a growth and education opportunity for the kids that was too important to pass up, and others said it enhanced the quality of the data they could collect. Ice et al. [45] indicate that having one’s family present can reduce loneliness, support research, and humanize researchers for a local community. As one participant stated,

having children is [not] a drag on academic careers. It can be precisely the opposite. My dissertation fieldwork would not have been nearly as rich without my family (husband and daughter). I have been tremendously productive. But each child is different and can cause vastly different challenges for travel.

Others pointed out that having children in the field made it more difficult because of caring for or worrying about their safety or that they avoided it so they could be productive. A 43-year-old Hispanic female student noted that “the option to bring my child wasn’t offered, and I didn’t want to risk my performance in the field worrying about the safety of my child.” A 43-year-old white male professional said, “I would not get any work done. Quality childcare is too expensive and it is not possible to conduct fieldwork while responsible for a child.” At a basic level, young children cannot be left alone while parents conduct fieldwork. As a result, parents must arrange for long-term care either at home or in the field. A spouse can provide this care, as can a family member, shared care services, or paid caregivers; but not all professionals have the same social and financial options for caregivers, and it is even rarer among students. Our findings indicate that individuals with a stay-at-home spouse or retired parent with a taste for adventure were more likely to be able to bring children to their field site than peers without these relationships. A 42-year-old white male professional put it:

I have conducted research that can accommodate my family life, which is ultimately more important to me than personal careerist aspirations. Fortunately for me, my wife and children are adventurous souls who, like me, are not necessarily looking for conventional middleclass security.

As many of our respondents had their first and second children before or during graduate school, social and emotional support for parents presents a critical feature in retaining diversity in anthropology.

### Family and career stress

Successfully managing the stress of a family and a career in a field-based discipline such as anthropology can be challenging. According to O’Laughlin and Bischoff [35], academics are subject to several types of family-career conflict, including time-based, strain-based, and behavior-based. The average academic, works approximately 55 hours/week balancing teaching, research, service, consultation, and other roles, which is a strain. Such strain is compounded when career and family roles are incompatible. These factors have been associated with individual health risks and depression in other studies [35,37]. Lack of family-career balance was the single most predictive influence on stress in all our models. Graduate students were especially likely to report imbalance associated with stress; this is consistent with high rates of anxiety and depression reported among academics [20].

In addition, several childless respondents indicated they had been discouraged from having children or had avoided it because they worried they would not be taken seriously, or because the demands of academia were perceived as too great a burden to expect balance. For instance, one respondent stated,

I am not a parent yet, though I do feel that the absolute command the PhD process has on my life is detrimental to the work/life balance needed to attend to other aspects of my life beyond meeting academic expectations. Instead of recognizing that this culture is damaging to students and faculty alike, and that striving to achieve a healthy work/life balance is important for long term productivity and career satisfaction, I have been advised to quit my program. I have been told that I don’t seem truly committed to the rigors of an academic life. I have been told that this is a demanding process and I should think about leaving if I want to strike a better balance between my personal needs and my professional advancement. I imagine this only gets worse once children come into the picture.

Social science professionals and students in general seem to know and fear a lack of family-career balance as a predictable tribulation of the discipline, which was suggested by our finding that students are already concerned about the impacts of family on getting tenure before they have even graduated or have a tenure-track position. As one 39-year-old male postdoc stated, “[I] want to give my child (and child to be due soon) sufficient attention. I don’t want to juggle pubs, teaching, and service for so little money and even less chance of success if it will negatively impact them.” A 39-year-old Asian-American female PhD student in our study said,

I don’t think having children would be perceived problematically by my peers. The biggest problem is that I have to make the wrong choices to remain in this work. I don’t face discrimination that I can discern because I already prioritize work over family and I take on more and more work, which leaves me little time for meaningful interactions with my kids. This is a huge problem and I’m not sure that the pressures of an academic career make me happy enough to sacrifice time with my family.

Yet, the relationship between family-career balance and psychosocial stress is not always negative, as remarked upon in other climate surveys of anthropology [23]. Although the financial rewards of becoming an anthropologist seem to diminish with each additional obstacle, anthropology as a career choice can also be exciting and rewarding in ways not measured by our survey. One respondent said that “[taking my child to the field] was difficult both for my child and me in terms of productivity, but it was a good experience overall.” Another stated, “a mixed experience for the children and I. Afterward a good experience, but difficult to see that during fieldwork.” For this reason, Halpern and colleagues suggest changing the metaphor to family-career “interactions”—balancing family and career in general is not a zero-sum game [8].

### Addressing barriers between women and fieldwork

Field-based data collection provides an extraordinary opportunity for comparative research. However, the demands of pursuing research away from home creates an expectation of socially unencumbered individuals who have the temporal, financial, and social resources to conduct this work. However, this perspective excludes the lived experience of anthropology professionals and trainees with social and financial obligations including (but not limited to) dependent children. Similar problems have been pointed out with regard to other field-based disciplines. In one study of gender issues reported by women in polar field research, 28% of respondents considered family commitments and caring issues an important source of inequality [40].

The intrinsic expectations of an anthropological field career can produce barriers to current and potential scholars. Our survey revealed that obstacles to anthropology as a field-based discipline are pervasive and multi-layered. Even people more likely to achieve success as field researchers in anthropology—those born into the relative wealth that provides socioeconomic privilege and support that continues while establishing a career and family—report high stress related to lack of balance between career and family. There are a number of family-friendly reforms for academia that have been implemented at progressive research institutions to address such issues, which others can adopt. In order of how commonly they’ve been implemented, reforms include six weeks paid maternity leave, maternal and dependent health insurance, stoppage of tenure-track clocks for mothers, modified duties for mothers after childbirth, college tuition remission for dependents, adoption expenses, lactation rooms, stoppage of tenure-track clocks for fathers, dual hires, subsidized childcare, modified duties for fathers, childcare grants for parents to attend conferences, emergency childcare, and part-time tenure-track appointments pre-and post-tenure [12]. Many of these benefits, especially parental leave after childbirth, should be entitlements that happen automatically, not privileges that must be applied for [12,16]. These benefits generally focus on research faculty, but, increasingly, awareness is spreading to liberal arts colleges and teaching institutions and extending to teaching faculty, adjuncts, graduate students, postdocs, and others in limited term positions.

Although some might assume that anthropology is more accepting of those with diverse life circumstances than other disciplines because of its study of human diversity, the limited existing evidence suggests that this is not the case. As Bassett writes, the “prevailing ethos of academic culture is that the career is to be prioritized over all else. To do otherwise is to risk being perceived as not committed to your profession, or worse, to risk not being taken seriously as a real scholar” [7]. Mason et al. [12] found that people within academia consider research universities hostile for faculty to necessities of family life. Higher education institutions frequently lack accountability for gender-related inequities [46,47], and the recent controversy about policing microaggressions [48] distracts from the systematic creation of spaces where safety is not evenly distributed. A 2016 survey of American Anthropological Association members affirms this, indicating that “women are significantly more likely than men to have experienced a hostile workplace and most types of unwanted sexual behaviors” and to report that their institution did not handle claims of sexual harassment in accordance with federal law [4]. These inequities apply as much to fieldwork as to campus life. Recently, two SAFE (Survey of Academic Field Experiences) studies within anthropology documented a high level of sexual harassment in field settings [6,49], a finding that has been replicated in other sciences [5]. These lines of investigation also highlight the lack of awareness among those with privilege that their tolerance of structural impediments imposes silence on those with less power [49]. In all these studies of discrimination and harassment, respondents felt that speaking out would jeopardize their careers in ways white men rarely experience [5,6,49,50].

Anthropologists must address these issues not only for ethical reasons, to advance our field, and to provide a model for other similar disciplines. Fieldwork is a critical practice that thickens and binds anthropology and renders it relevant for explaining human complexity. In training and experience, anthropologists are uniquely situated to compare culture and identify social injustice in the world. Yet struggles with intersectionality among anthropologists make our expertise suspect. Only by addressing the access and socialization within anthropology and other field-based disciplines will it begin to reflect those it claims to represent.

### Study limitations

One of the limitations of this study is associated with the use of an online self-report survey. Language used in recruitment may have introduced bias consistent with our hypothesis; however, responses spanned a continuum of agreement/disagreement. Additionally, recognizing our inability to anticipate all potential answers, we encouraged participants to provide comments throughout, enabling them to describe their unique circumstances. This was particularly important due to our unintended bias towards a nuclear family model. Because of our survey’s anonymity and public availability, we had no mechanism to prevent individuals from taking it multiple times though we have no reason to suspect that is the case.

Additionally, convenience surveys are inherently limited in that they provide only imperfect evidence, but we believe our survey provided enough data to establish that there are issues within the discipline of anthropology that departments and professional organizations should take measures to address. For instance, although we attempted to recruit all working or graduate student anthropologists regardless of experience or family circumstances, we repeatedly heard that people thought the survey was only for parents or those who took children into the field, which may have skewed the results. We also took steps to increase our recruitment of non-white participants but had lower responses than anticipated.

Furthermore, though we examine associations among family-career balance, stress, and career status, we frame our discussion as though there is linear causality, such that, for professionals at least, family-career balance seems to moderate how stressful one’s career status is. Though this pathway reflects findings from other studies [35], we acknowledge that there are as many possible causality pathways as there are intersections among respondents. For example, it is possible and implied by many qualitative respondents’ comments that some adjust their family-career balance till stress is sufficiently minimized to be personally tolerable.

Finally, our total sample included higher proportions of women and sociocultural anthropologists than other groups and overrepresented younger respondents in spite of the fact that we reached out purposefully to all genders, subdisciplines, and ages. The higher numbers of women and sociocultural anthropologists may be due to a greater interest in the study topic or to the larger numbers of sociocultural anthropologists in the discipline and of women among younger anthropologists [3]. However, we did have equal numbers of men among professionals and students. Regular social media users tend to be younger than people who primarily use email [51], so it is likely that our sampling skewed toward younger anthropologists. Furthermore, though we had equal numbers of professionals and students, those with strong feelings of family-career imbalance may be principally students and relatively younger professionals in the prime childrearing period of their lives.

This paper represents the first steps in exploring these data. Future articles will explore the role of ethnicity, status of first-generation college students in accessing an anthropological career, and how anthropology fares in supporting breastfeeding and maternal and paternal leave, among other workplace issues.

## Conclusions and future

The majority of our respondents were white and from college-educated US households—the demographic group most likely to have access to resources that allowed them to succeed at the highest levels of education [15,17,52]. Yet, the transition from graduate school to permanent employment is precarious, especially for women [17,53]. We confirmed that, in anthropology, white men were more likely to become tenured professors than women or minority men. Furthermore, our study found that expectations of an anthropology career influenced family planning decisions for both women and men; however, impacts were greater for women. The biggest concerns for participants were, depending on model construction, being able to conduct fieldwork and have children and aspects of stable employment.

We found that family-career balance was the most significant predictor of stress for both professional and graduate students. High stress perception was pervasive, especially among students. Younger professionals reported significantly higher stress related to family-career imbalance relative to older respondents, and students self-reporting as lower SES had higher stress. Combined, our findings are similar to those of other STEM and related fields and suggest that field-based disciplines like anthropology may be self-limiting because of socioeconomic factors associated with gender, class, ethnicity, and other personal factors, which may ultimately undermine the integrity of these disciplines and their constructions of knowledge. We take heart, therefore, that education and gender equality are integral parts of the UN 2030 Agenda for sustainable development adopted by the United Nations General Assembly in 2015 and the statement that holds as true for anthropology: “Girls and women are key players in crafting solutions to improve lives…They are the greatest untapped population to become the next generations of STEM professionals—we must invest in their talent” [43]. Based on the findings of this study, investing in their talent means taking an active role in supporting family career balance.

## Supporting information

S1 Appendix The survey that we administered is appended. This is the second iteration of the survey, which includes items querying socioeconomic status. The survey was administered using Qualtrics and included skip logic, which skipped respondents past questions that did not apply to them, based on previous answers. However, items numbers were not retained when copying the survey, so skip logic markers refer to numbers that are not visible in this supplement. Furthermore, some sections would not have been visible to some respondents—for instance, the section on childcare in the field would not be visible to respondents with no children.
(DOCX)
