# Supplementary material for: Family and the field: Expectations of a field-based research career affect researcher family planning decisions
Source: PLoS One. 2018 Sep 7;13(9):e0203500. doi: 10.1371/journal.pone.0203500 (PMC6128561; doi:10.1371/journal.pone.0203500)
Supplement: S1 Appendix — This is the second iteration of the survey, which includes items querying socioeconomic status. The survey was administered using Qualtrics and included skip logic, which skipped respondents past questions that did not apply to them, based on previous answers. However, items numbers were not retained when copying the survey, so skip logic markers refer to numbers that are not visible in this supplement. Furthermore, some sections would not have been visible to some respondents—for instance, the section on childcare in the field would not be visible to respondents with no children. (DOCX) [file pone.0203500.s001.docx]

## S1 Appendix

### Family & the Field - II

We are investigating the challenges associated with managing a fieldwork-oriented career path and parenting responsibilities or family planning. This study provides the opportunity for us as anthropologists to turn our gaze inwards. The goal of this study is to examine the pressures, decisions, and compromises that field-based anthropologists make in deciding to do fieldwork, have a family, or to navigate doing both simultaneously.

In anthropology, fieldwork is frequently conceptualized as a pursuit conducted by socially and financially unencumbered individuals. However, this idealized narrative of the “lone researcher” may or may not reflect the experiences of working anthropologists or students in anthropology. This “lone researcher” model can also be contrasted with that of the anthropologists who take their offspring to the field and raise “worldly children,” with the costs and other encumbrances of such experiences scarcely mentioned.

We seek to investigate the actual professional and graduate student attitudes associated with parenthood in anthropology. The following survey queries how you, as professionals and graduate students in anthropology, may negotiate academia to accommodate parenthood and how cultural models of parenthood and fieldwork may have shifted over time.

**DEMOGRAPHIC INFORMATION**

This section queries basic demographic information and your general background as an anthropologist.

I identify as:

- Female
- Male
- Other: ________________________________________________

| 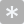 |
| --- |

What is your current age?

________________________________________________________________

Skip To: D3 If What is your current age? >= 18

| 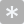 |
| --- |

What is your birth date? Enter in mm/dd/yyyy format.

________________________________________________________________

How would you most accurately describe your current civil status?

- Single
- Separated/divorced/widowed
- In a committed relationship
- Married

Skip To: D5 If How would you most accurately describe your current civil status? = Single

Skip To: D5 If How would you most accurately describe your current civil status? = Separated/divorced/widowed

Does your partner have an academic position?

- No
- Yes

What country or nation were you RAISED in?

________________________________________________________________

What country or nation do you CURRENTLY LIVE in?

________________________________________________________________

What racial/ethnic/heritage group do you typically identify or classify yourself with (e.g., white, black, Hispanic, Asian, American, etc.)?

________________________________________________________________

Which of the following BEST describes you and your background?

- First generation college student
- Parents received some college education
- One or both parents completed bachelor’s as highest degree
- One or both parents completed master’s as highest degree
- One or both parents completed doctorate as highest degree

Which of these BEST reflects your current employment/position?

- Master’s-level Graduate Student
- Doctoral-level Graduate Student
- Post-Doc
- Adjunct Professor
- Full Time Lecturer
- Assistant Professor
- Associate Professor
- Full Professor
- Emeritus

What is the highest degree you have completed?

- Bachelor degree
- Master’s degree
- Doctoral degree
- Other: ________________________________________________

Which of the following best describes your current circumstances?

- Not applicable (e.g., I’m a student)
- Unemployed
- Underemployed
- Fully-employed
- Other: ________________________________________________

Indicate the highest education completed by your mother figure, your father figure, and your spouse/partner. If you grew up in a single caregiver home, indicate only the answer for your one caregiver. (You will be given the opportunity later in this survey to clarify this.) If you neither married nor partnered, do not answer for that item.

|  | Less than 7th grade | Junior high / Middle school (9th grade) | Partial high school (10th or 11th grade) | High school graduate | Partial college (at least one year) | College education | Graduate degree |
| --- | --- | --- | --- | --- | --- | --- | --- |
| Mother figure |  |  |  |  |  |  |  |
| Father figure |  |  |  |  |  |  |  |
| Spouse/partner |  |  |  |  |  |  |  |

Indicate which of the following most accurately describes the occupation of your mother figure, father figure, and spouse/partner. If you grew up in a single-parent home, answer only for that parent. If you are not married or partnered, answer only for your parent figures.

|  | Day laborer, janitor, house cleaner, farm worker, food counter sales, food preparation worker, busboy. | Garbage collector, short-order cook, cab driver, shoe sales, assembly line workers, masons, baggage porter. | Painter, skilled construction trade, sales clerk, truck driver, cook, sales counter or general office clerk. | Automobile mechanic, typist, locksmith, farmer, carpenter, receptionist, construction laborer, hairdresser. | Machinist, musician, bookkeeper, secretary, insurance sales, cabinet maker, personnel specialist, welder. | Supervisor, librarian, aircraft mechanic artist and artisan, electrician, administrator, military enlisted personnel, buyer. | Nurse; skilled technician; medical technician; counselor; manager; police and fire personnel; financial manager; physical, occupational, speech therapist. | Mechanical, nuclear, and electrical engineer; educational administrator; veterinarian, military officer; elementary, high school and special education teacher. | Physician, attorney, professor, chemical and aerospace engineer, judge, CEO, senior manager, public official, psychologist, pharmacist, accountant. |
| --- | --- | --- | --- | --- | --- | --- | --- | --- | --- |
| Mother figure |  |  |  |  |  |  |  |  |  |
| Father figure |  |  |  |  |  |  |  |  |  |
| Spouse/partner |  |  |  |  |  |  |  |  |  |

Think of this ladder with 9 steps as representing where people stand in the country where you grew up. At the top are people who are the best off--those with the most money, most education, and most respected jobs. At the bottom are the people who are worst off--those who have the least money, least education, and the least respected jobs or no job. Where would you place yourself on this ladder?

| 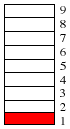 | 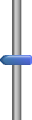 |  |
| --- | --- | --- |

| 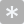 |
| --- |

How many YEARS did it take for you to complete your highest-obtained degree?

________________________________________________________________

Compared to others in your final graduate program, what was your time to completion?

- Slower than others
- Average
- Faster than others

Which anthropological subfield(s) were you principally trained in?

- Applied anthropology
- Archaeology
- Biological anthropology
- Cultural anthropology
- Linguistic anthropology
- Other: ________________________________________________

Which of these describes where you have conducted research? (Mark all that apply) I conducted research in:

|  | First project | 2nd project | 3rd project | 4th project | 5th project | 6th project | 7th project | Additional projects |
| --- | --- | --- | --- | --- | --- | --- | --- | --- |

| North America |  |  |  |  |  |  |  |  |
| --- | --- | --- | --- | --- | --- | --- | --- | --- |
| Central or South America |  |  |  |  |  |  |  |  |
| Europe |  |  |  |  |  |  |  |  |
| Asia |  |  |  |  |  |  |  |  |
| Africa |  |  |  |  |  |  |  |  |
| Oceania |  |  |  |  |  |  |  |  |
| Other: |  |  |  |  |  |  |  |  |
| Not applicable—I did not conduct research |  |  |  |  |  |  |  |  |

In the last month, how often have you felt that you were unable to control the important things in your life?

- Never
- Almost never
- Sometimes
- Fairly often
- Very often

| 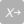 |
| --- |

In the last month, how often have you felt confident about your ability to handle your personal problems?

- Never
- Almost never
- Sometimes
- Fairly often
- Very often

In the last month, how often have you felt that things were going your way?

- Never
- Almost never
- Sometimes
- Fairly often
- Very often

In the last month, how often have you felt difficulties were piling up so high that you could not overcome them?

- Never
- Almost never
- Sometimes
- Fairly often
- Very often

How would you rank your family life and career/school balance?

- Terrible
- Poor
- Acceptable
- Good
- Excellent

Do you have any children (biological, step, adopted, or otherwise)?

- No
- Yes

Skip To: End of Block If Do you have any children (biological, step, adopted, or otherwise)? = No

Indicate the number of children you have who are:

|  | 1 | 2 | 3 | 4 | 5 | 6 | 7 | 8 | 9 | 10+ |
| --- | --- | --- | --- | --- | --- | --- | --- | --- | --- | --- |
| Biological children |  |  |  |  |  |  |  |  |  |  |
| Adopted children |  |  |  |  |  |  |  |  |  |  |
| Foster children |  |  |  |  |  |  |  |  |  |  |
| Total children |  |  |  |  |  |  |  |  |  |  |

How many children do you have of the following ages?

|  | 1 child | 2 children | 3 children | 4 children | 5 or more children |
| --- | --- | --- | --- | --- | --- |

| Under 1 year |  |  |  |  |  |
| --- | --- | --- | --- | --- | --- |
| 1 year to 4 years 11 months |  |  |  |  |  |
| 5 years to 11 years 11 months |  |  |  |  |  |
| 12 years to 14 years 11 months |  |  |  |  |  |
| 15 years to 18 years 11 months |  |  |  |  |  |
| 19 years to 20 years 11 months |  |  |  |  |  |
| 21 years or older |  |  |  |  |  |

**FUNDING IN GRADUATE SCHOOL** This section inquires about the general level of support you received or are currently receiving for graduate study in anthropology, irrespective of your parenthood status or plans.

How did/are you paying for the majority of graduate school? (Choose all that apply.)

- Financial support from spouse, family, or friends
- Personal savings
- Part-time work outside my department
- Full-time work outside my department
- Minimal support from my department
- Partial support from my department with teaching or research expectations
- Partial support from my department without teaching or research expectations
- Full support from my department with teaching or research expectations
- Full support from my department without any teaching or research expectations
- Other: ________________________________________________

Did/do you receive a living stipend from your graduate school support sufficient for the following (select all that apply)?

- Begin degree program?
- Partially complete degree program?
- Finish degree program?

How did your funding compare to that of peers at the same level in your final graduate program (the one from which you received or will receive your ultimate degree)? Was/is your funding:

- Less than average
- About average
- More than average

**YOUR PARENTS / CAREGIVERS**
This section inquires about what kind of role models you had for parenting when you were growing up. In the following sections, we would like you to expand on the influences family and career options and opportunities have had on your family-career balance. In several cases, we have done this by asking a question for which the answers are categorical (i.e., we give you several choices). However, you may not fit exactly in the choices or would like to explain how the choices apply to you. We have provided text boxes under those questions to allow you to expand on your answers and encourage you to do so. As you know, details will improve the quality of the data we are able to collect.

Who was/were your primary caregiver(s) growing up? (Mark ALL that apply.)

- Mother(s)
- Father(s)
- Stepfather
- Stepmother
- Grandmother
- Grandfather
- Aunt
- Uncle
- Godparent(s)
- Other: ________________________________________________

How engaged was/were your primary caregiver(s) WITH HER/HIS WORK OUTSIDE THE HOME when you were growing up? If you had more than one primary caregiver (e.g., a mother and father), you may want to check a different box for each person.

|  | Did not work | Worked part-time | Worked full-time | Career-oriented | Very career-oriented |
| --- | --- | --- | --- | --- | --- |

| Mother |  |  |  |  |  |
| --- | --- | --- | --- | --- | --- |
| Father |  |  |  |  |  |
| Other |  |  |  |  |  |
| Other |  |  |  |  |  |

How accessible was/were your primary caregiver(s) to you AS A PARENT when you were growing up? Check a box for each caregiver. If all primary caregivers were equally accessible, check one box.

|  | Not accessible at all | Somewhat accessible | Relatively accessible | Very accessible |
| --- | --- | --- | --- | --- |

| Mother |  |  |  |  |
| --- | --- | --- | --- | --- |
| Father |  |  |  |  |
| Other |  |  |  |  |
| Other |  |  |  |  |

**FAMILY PLANNING**

The following section queries your potential parenting plans. There are many influences on family planning that cannot reasonably be accommodated via a simple questionnaire. Please help us flesh out this complexity by providing explanations for your answers where it is relevant.

What are your future parenting plans for biological or adopted child(ren)?

- I will not have children
- It is unlikely that I will choose to have children
- I am unsure if I want to become a parent
- I plan on becoming a parent

Please explain:

________________________________________________________________

________________________________________________________________

________________________________________________________________

________________________________________________________________

________________________________________________________________

If I have a child(ren), I will likely

- Leave my school/job to take care of my child
- Continue working but curtail the amount of time I invest in career
- Attempt to achieve a balance between family life and career
- Work until I retire
- Work for the rest of my life
- Undecided / unsure
- Not applicable

Please explain:

________________________________________________________________

________________________________________________________________

________________________________________________________________

________________________________________________________________

________________________________________________________________

Do you feel you are/were treated different at work/school by the following because you are/were NOT a parent? (Select ALL that apply.)

|  | No | Yes, they treated me with MORE respect | Yes, they avoided or ignored me | Yes, they treated with LESS respect | Other POSITIVE treatment | Other NEGATIVE treatment | Not applicable |
| --- | --- | --- | --- | --- | --- | --- | --- |

| Colleagues |  |  |  |  |  |  |  |
| --- | --- | --- | --- | --- | --- | --- | --- |
| Supervisor |  |  |  |  |  |  |  |
| Adviser |  |  |  |  |  |  |  |
| Committee members |  |  |  |  |  |  |  |
| Fellow students |  |  |  |  |  |  |  |
| Others: |  |  |  |  |  |  |  |

Does your current employer offer sabbaticals or other family leave opportunities for people in your position (beyond those mandated by the state)?

- No
- Unsure
- Yes

How does/would the presence of sabbaticals or other family leave opportunities impact your motivation regarding the following?

|  | Decrease(s) motivation | No effect on motivation | Increase(s) motivation |
| --- | --- | --- | --- |
| Remain in your program / career |  |  |  |
| Start a family |  |  |  |

What influence did the expectations or responsibilities of a career in anthropology have on your decision(s) to put off or not have a family?

- No influence at all
- A little influence
- Substantial influence
- Tremendous influence

Skip To: FP6 If What influence did the expectations or responsibilities of a career in anthropology have on your... = No influence at all

Which expectations of an anthropological career impact(ed) your decision(s) to put off or not have a family? Please choose all that apply.

- Salary constraints
- Challenges with taking children when conducting fieldwork
- Wouldn’t be able to do all the things needed to earn tenure
- Impact promotion
- Vertical and horizontal social pressure
- Preconceived notions of peers
- Concerns associated with disappointing advisers
- Concerns associated with disappointing colleagues
- Concerns associated with disappointing family
- Perceived loss of professional legitimacy
- Not applicable

Which of these pressures have you experienced as a non-parent from those within your department and/or discipline?

- Assumptions that schedule more flexible than those with children
- Assumptions that you have more free time than those with children
- Assumptions that you have more money than those with children
- Assumption that you should be more productive than those with children
- Assumption that you should take on more service requirements then those with children
- General pressures / stigmas /expectations associated with being a non-parent

**CHILDREN AND YOUR CAREER**

This section inquires about your status as a parent and family composition.

What career stage were you at when you had or adopted your child(ren)?

|  | Before graduate school | During graduate school | Between graduate school and post-graduate position (post-doc, visiting professor, tenure-track) | During limited position (post-doc, visiting professor, adjunct, etc.) | Within 1st two years of long-term contractual employment (tenure-track, lecturer, etc.) | Pre-tenure | Post-tenure |
| --- | --- | --- | --- | --- | --- | --- | --- |

| 1st child |  |  |  |  |  |  |  |
| --- | --- | --- | --- | --- | --- | --- | --- |
| 2nd child |  |  |  |  |  |  |  |
| 3rd child |  |  |  |  |  |  |  |
| 4th child |  |  |  |  |  |  |  |
| 5th child |  |  |  |  |  |  |  |
| 6th child |  |  |  |  |  |  |  |
| 7th child |  |  |  |  |  |  |  |
| 8th child |  |  |  |  |  |  |  |
| 9th child |  |  |  |  |  |  |  |
| Any additional children |  |  |  |  |  |  |  |

Which of the following best describes the planning of your biological children?

- Unplanned
- Planned
- One or more were planned, one or more unplanned

Which of these best describes your parental status?

- Single parent
- Step-parent
- Two-parent nuclear family
- Shared custody
- Other: ________________________________________________

Skip To: C6 If Which of these best describes your parental status? != Single parent

Are you currently a single parent of a child(ren) under the age of 21?

- No
- Yes, with custody of my child(ren)
- Yes, with joint custody of my child(ren)
- Yes, but I don’t have custody or joint custody of my child(ren)

How did you become a single parent?

- Unmarried when child was born
- Unmarried when child was adopted
- Divorce death of spouse
- Other: ________________________________________________

Do you support or contribute to the financial support of any of your child(ren)?

- No
- Yes

Which of these best describes your financial obligations to your child(ren)?

- None
- Partial (Less than 25%)
- Partial (Between 25-50%)
- Majority (Between 50-75%)
- Majority (Between 75-100%)
- Other ________________________________________________

**FIELD EXPERIENCE WITH CHILDREN**
The following questions ask about the logistical considerations of managing young children while conducting fieldwork.

Have you been to the field since giving birth/adopting?

- No
- Yes

Skip To: FE5 If Have you been to the field since giving birth/adopting? = No

How frequently do you go to a field site that requires travel away from your home for multiple overnight stays?

- Never
- Once ever
- A few times in my career
- Every few years or so
- Annually
- Multiple times per year

Please list the length of the periods of time you have spent in the field and whether or not you were a parent when you were there:

|  | Length of time in field | Parent at time? | |
| --- | --- | --- | --- |
|  | Months | No | Yes |
| First fieldwork experience: |  |  |  |

| 2nd fieldwork experience: |  |  |  |
| --- | --- | --- | --- |
| 3rd fieldwork experience: |  |  |  |
| 4th fieldwork experience: |  |  |  |
| 5th fieldwork experience: |  |  |  |
| 6th fieldwork experience: |  |  |  |
| 7th fieldwork experience: |  |  |  |
| 8th fieldwork experience: |  |  |  |
| 9th fieldwork experience: |  |  |  |
| Additional fieldwork experiences: |  |  |  |

Do or have you ever taken your minor child(ren) to a field site you work at that is far away from home while you conducted research?

- Never
- Once or a few times
- More often than not
- Always or almost always

Why or why not?

________________________________________________________________

________________________________________________________________

________________________________________________________________

________________________________________________________________

________________________________________________________________

Which of the following best describes your fieldwork experience with your child(ren) along?

- Not applicable (I've never taken them with me)
- Unproductive/mistake for the child(ren) and/or me
- Good for the child(ren) but difficult in some way (financially, productively)
- Difficult for the child(ren) but productive for my research
- Good overall experience for my child(ren) and productive for me
- Other: ________________________________________________

Who usually cares for your child(ren) when you are away from home overnight for the following?

|  | Co-parent | A grandparent | A non-grandparent relative | A non-relative | I take my children with me |
| --- | --- | --- | --- | --- | --- |
| In the field |  |  |  |  |  |
| At conferences/workshops? |  |  |  |  |  |
| Work-related events besides fieldwork and conferences? |  |  |  |  |  |

When you are doing fieldwork or working away from home overnight, does your child(ren) have to temporarily move into a home other than their own?

- No
- Yes

**INSTITUTIONAL SUPPORT FOR PARENTING** 
This section inquires about the amount of institutional support you received or are currently recently to balance parenting and work.

How supportive of your academic/career efforts is/was the following while you were raising child(ren)?

|  | Not supportive at all | Somewhat supportive | Supportive | Very supportive |
| --- | --- | --- | --- | --- |
| Partner |  |  |  |  |
| Academic colleagues |  |  |  |  |
| Academic advisor |  |  |  |  |
| Department chair (if applicable) |  |  |  |  |
| Employer (dean or other ultimate supervisor) |  |  |  |  |

Please explain:

________________________________________________________________

________________________________________________________________

________________________________________________________________

________________________________________________________________

________________________________________________________________

How supportive is the institution you are/were affiliated with regarding parental responsibilities (e.g., maternity/paternity leave, childcare, paid time for sick children, vacation accrual, flexible scheduling)?

- Penalties for missing activities due to parental responsibilities
- No penalties but no institutional support or services
- Some institutional support (e.g., maternity/paternity leave, childcare, paid time for sick children, vacation accrual, flexible scheduling)
- Significant institutional support and programming for employees/students with children

If you are/were in a tenure track position, are/were you able to stop the tenure clock?

- No
- Unsure
- Yes
- Not applicable

Skip To: End of Block If If you are/were in a tenure track position, are/were you able to stop the tenure clock? = No

Skip To: End of Block If If you are/were in a tenure track position, are/were you able to stop the tenure clock? = Unsure

Skip To: End of Block If If you are/were in a tenure track position, are/were you able to stop the tenure clock? = Not applicable

Did you choose to stop the tenure clock?

- No
- Yes

**FAMILY – CAREER BALANCE**

To what extent did or has becoming a parent motivated you to change your career path?

- Not a factor at all
- Small factor in decision
- Big factor in decision
- Main motivation to change

Which expectations of an anthropological career have impacted your decisions about having a family and the timing of those decisions. (Mark ALL that apply.)

- Salary constraints
- Challenges with taking children when conducting fieldwork
- Wouldn’t be able to do all the things needed to earn tenure
- Impact promotion
- Vertical and horizontal social pressure
- Preconceived notions of peers
- Concerns associated with disappointing advisers
- Concerns associated with disappointing colleagues
- Concerns associated with disappointing family
- Perceived loss of professional legitimacy
- Other: ________________________________________________

What is/was the gender of your direct supervisor at work when you were raising children? Please select all that apply if you have had multiple direct supervisors while raising your children.

- Female
- Male
- Other: ________________________________________________

Skip To: FC4 If Selected Choices < 2

Please explain your answer if you had more than one direct supervisor:

________________________________________________________________

Did you feel you were treated different at work/school by the following because you became a parent? (Select ALL that apply.)

|  | No | Yes, they treated me with MORE respect | Yes, they avoided or ignored me | Yes, they treated with LESS respect | Other POSITIVE treatment | Other NEGATIVE treatment | Not applicable |
| --- | --- | --- | --- | --- | --- | --- | --- |

| Colleagues |  |  |  |  |  |  |  |
| --- | --- | --- | --- | --- | --- | --- | --- |
| Supervisor |  |  |  |  |  |  |  |
| Adviser |  |  |  |  |  |  |  |
| Committee members |  |  |  |  |  |  |  |
| Fellow students |  |  |  |  |  |  |  |
| Others: |  |  |  |  |  |  |  |

Did you take any time off (Family Medical Leave Act or otherwise) with the birth of your child(ren)?

|  | Took time off after birth of | | Time taken off | |
| --- | --- | --- | --- | --- |
|  | No | Yes | Years | Months |
| First child |  |  |  |  |
| Second child |  |  |  |  |
| Third child |  |  |  |  |
| Fourth child |  |  |  |  |
| Fifth child |  |  |  |  |
| Sixth child |  |  |  |  |
| Seventh child |  |  |  |  |
| Eighth child |  |  |  |  |
| Ninth child |  |  |  |  |
| Additional child(ren) |  |  |  |  |

**MOTHERHOOD AND ACADEMIA**

This section inquires specifically about breastfeeding children while maintaining a career or job.

Did/do you breastfeed or pump breast milk to feed your child(ren) after delivery?

- No
- Breastfed one child only
- Breastfed multiple children

If this varied for multiple children, please explain:

________________________________________________________________

________________________________________________________________

________________________________________________________________

________________________________________________________________

________________________________________________________________

How many months did you **plan** to breastfeed or pump milk to feed your child(ren)?

- Less than 1 month
- __ months *(indicate number of months in box below)* ________________________________________________

If this varied for multiple children, please explain:

________________________________________________________________

________________________________________________________________

________________________________________________________________

________________________________________________________________

________________________________________________________________

How many months did you **actually**breastfeed or pump milk to feed your child(ren)?

- Less than 1 month
- __ months *(indicate number of months in box below)* ________________________________________________

If this varied for multiple children, please explain:

________________________________________________________________

________________________________________________________________

________________________________________________________________

________________________________________________________________

________________________________________________________________

Were you breastfeeding or pumping when you returned to work/school?

- No
- Yes

If this varied for multiple children, please explain:

________________________________________________________________

________________________________________________________________

________________________________________________________________

________________________________________________________________

________________________________________________________________

Were you given time at work/school to pump your breasts or breastfeed your baby?

- No
- Yes

If this varied for multiple children, employers, or schools, please explain:

________________________________________________________________

________________________________________________________________

________________________________________________________________

________________________________________________________________

________________________________________________________________

Were you given a comfortable, sanitary, and private location for breastfeeding or pumping at work/school?

- No
- Yes
- Other: ________________________________________________

If this varied for multiple children, employers, or schools, please explain:

________________________________________________________________

________________________________________________________________

________________________________________________________________

________________________________________________________________

________________________________________________________________

If you pumped, were you provided a sanitary location to store breast milk?

- No
- Yes
- Other: ________________________________________________

If this varied for multiple children, employers, or schools, please explain:

________________________________________________________________

________________________________________________________________

________________________________________________________________

________________________________________________________________

________________________________________________________________

Did you stop breastfeeding earlier than you had initially intended to because of work/school?

- No
- Yes

Skip To: End of Block If Did you stop breastfeeding earlier than you had initially intended to because of work/school? = No

What were your reasons for stopping breastfeeding before you planned related to work/school? (Mark ALL that apply.)

- Does not apply; I did not stop breastfeeding because of work
- I didn’t think my supervisor would give me time to breastfeed or pump at work
- My supervisor said s/he would not give me time to breastfeed or pump at work
- The time I could use to pump needed to be devoted to something else (lunch, studying, grading, meeting with students, etc.)
- My co-workers wouldn’t support my pumping
- There wasn’t any place for me to pump in my work area
- I didn’t have a breast pump
- I couldn’t store my breast milk
- Other work/school reason _____ ________________________________________________

If this varied for multiple children, employers, or schools, please explain:

________________________________________________________________

________________________________________________________________

________________________________________________________________

________________________________________________________________

________________________________________________________________

End of Block: Motherhood & Academia

**FINAL THOUGHTS**

The final section requests your comments and contact information for follow-up investigation, if you are willing or interested.

We realize a questionnaire limits our capacity to assess the diversity of parenting experiences among anthropologists. Please share any comments you have on parenthood and anthropology or parenthood and the field that you do not feel have been addressed here or would like to comment further on and consider allowing us to contact you for a follow-up interview.


Comments:

________________________________________________________________

________________________________________________________________

________________________________________________________________

________________________________________________________________

________________________________________________________________

May we contact you with follow-up questions?

- No
- Yes

Skip To: End of Survey If May we contact you with follow-up questions? = No

Would you prefer to be contacted by email or phone?

- Email
- Phone

Skip To: FT2d If Would you prefer to be contacted by email or phone? = Phone

| 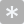 |
| --- |

What is your email address?

________________________________________________________________

Skip To: End of Survey If What is your email address? Is Not Empty

| 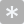 |
| --- |

What is your phone number?

________________________________________________________________
